# A Simple and Safe Technique for CT Guided Lung Nodule Marking prior to Video Assisted Thoracoscopic Surgical Resection Revisited

**DOI:** 10.1155/2015/235720

**Published:** 2015-10-22

**Authors:** James A. Stephenson, Ayman Mahfouz, Sridhar Rathinam, Apostolos Nakas, Amrita Bajaj

**Affiliations:** ^1^Department of Radiology, Glenfield General Hospital, University Hospitals of Leicester, Groby Road, Leicester LE3 9QP, UK; ^2^Department of Thoracic Surgery, Glenfield General Hospital, University Hospitals of Leicester, Groby Road, Leicester LE3 9QP, UK

## Abstract

*Aim*. We describe our experience of a simple, safe, and reproducible technique for lung nodule marking prethoracoscopic metastasectomy. Thoracoscopic lung nodule resection reduces patient discomfort, complications, higher level of care, hospital stay, and cost; however, small deeply placed lung nodules are difficult to locate and resect thoracoscopically. *Materials and Methods*. We describe and review the success of our novel technique, where nodules are identified on a low dose CT and marked with methylene blue using CT fluoroscopy guidance immediately prior to surgery.* Results*. 30 nodules were marked with a mean size of 8 mm (4–18 mm) located at a mean depth of 17 mm, distributed through both lungs. Dye was detected at the pleural surface in 97% of the patients and at the nodule in 93%. There were no major complications. Thoracoscopic resection was possible in 90%.* Conclusion*. This is a simple and safe method of lung nodule marking to facilitate thoracoscopic resection in cases where this may not be technically possible due to nodule location.

## 1. Introduction

Video Assisted Thoracic Surgery (VATS) was introduced in the early 1990s [1, 2] and is now widely considered to be the gold standard for suspected malignant lung nodule resection. The technique might reduce patient discomfort, complications, the need for higher level of care, hospital stay, and cost compared to open thoracotomy. In 1999, a review of VATS lung nodule resection demonstrated that 54% of the cases required conversion to open thoracotomy to complete resection. The study identified that nodule localization failure was the cause for the majority of conversions (46%) [3]. The challenge increases further with small deeply placed lung nodules as the visceral pleura may not demonstrate any altered shape or colour, making these nodules even more difficult to locate thoracoscopically [4].

A number of differing methods of preoperative “marking” have been reported in the literature with varying degrees of success, complications, cost, specialised equipment, and required expertise. We revisit a simple but safe technique using computed tomography (CT) guided marking using methylene blue injection prior to VATS pulmonary metastasectomy described in both the adult and the paediatric populations dating back to the 1990s [5, 6] but subsequently underutilized.

## 2. Method

Using our technique, nodules are identified on a “low dose” CT (120 kV, modulated mA and 6.0 mm acquisitions) prior to marking. A metallic skin marker is used to aid in localisation and to plan an appropriate cutaneous needle insertion point. To dilute the methylene blue safely, 5 mL of the patients' venous blood is withdrawn and mixed with methylene blue aseptically in a ratio of 1 part methylene blue to 5 parts autologous blood.

**Figure 1:**
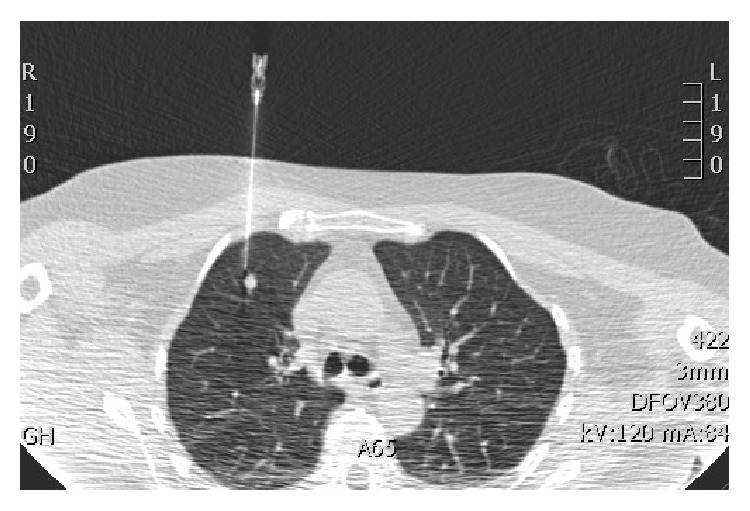
Image capture from CT fluoroscopy: needle inserted under CT fluoroscopic guidance to a position adjacent to the target nodule.

**Figure 2:**
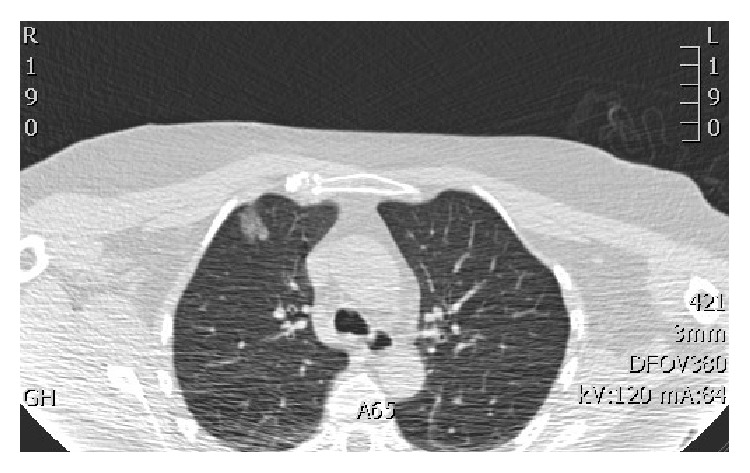
Image capture from CT fluoroscopy: the needle has been retracted and removed. The high-density methylene blue/blood track to the pleural surface can be identified.

Then, under local anaesthesia and aseptic conditions, the nodule is then marked using CT fluoroscopy. In our institute, we use manually controlled pulsed CAREVision (Siemens Somatom) CT fluoroscopic guidance (120 kV and a mAs of 30). The nodule is approached with a 16/18G spinal needle in a way akin to lung biopsy (Figure 1). On reaching the nodule, the methylene blue blood mixture is injected immediately adjacent to the nodule and along the needle tract right up to the pleural surface as the needle is retracted, leaving a methylene blue/blood tract (Figure 2).

The procedure is performed in the radiology department immediately prior to surgery. Once in the operating department, the surgeon initially reviews the coronal reformatted images and can then locate the dye on the pleural surface under direct vision using the thoracoscope, guiding the approach to the parenchymal nodule.

When the needle is placed slightly superior or inferior to the nodule due to technical difficulties, such as the approach being hindered by overlying ribs, the dye may not be immediately adjacent to the nodule. In this case, CT reconstructions and dialogue with the operating surgeon are essential.

## 3. Results

We have marked 30 nodules over 2 years. The mean patient age was 68 (51–81), with a BMI of 25 (22–28) and a mean FEV1 98% of predicted (82–115%). The mean nodule size was 8 mm (4–18 mm) located at a mean depth of 17 mm (6–42 mm), distributed through both lungs (right = 17 (upper = 5, lower = 12) and left 13 (upper = 8, lower = 5)). The mean volume of dye injected was 3 mL (2–4 mL). Dye was detected at the pleural surface in 97% (29/30) of the patients and at the nodule in 93% (28/30). There were no major complications, but two small pneumothoraces with no clinical significance, and one patient suffered discomfort; this was resolved with secondary local anaesthetic injection. There was no adverse effect on general anesthesia, and thoracoscopic resection was possible in 90% (27/30) of the cases. One of the cases converted to open resection which was due to technical difficulties with the surgery rather than being related to the marking. In the other two cases in which thoracoscopic resection was not possible the location of the nodule made it difficult to ensure complete resection with VATS resection, and this only became apparent during the surgery.

## 4. Discussion

Computed tomography guided radiological marking techniques described include wire marking [7, 8], lipiodol marking [9], barium marking [10], and combinations of wire and lipiodol marking [11]. CT guided wire markings have been associated with uncontrollable pneumothorax and wire dislodgement, as well as high proportion of minor adverse events such as small pneumothorax and perifocal bleeding [7, 8, 11, 12]. The technique, using CT guided administered barium contrast balls to mark lung nodules, was found to cause local acute inflammations in all cases [10], and this can potentially hinder surgical resection.

The use of StealthStation Treon treatment guidance system (Medtronic; Louisville, KY) has been described. The system navigates the administration of methylene into or adjacent to the lung nodule. Results have been successful with no reported major complications; however, the equipment can be costly and requires training for use [13].

Surgical techniques using methylene blue have been described with the surgeon palpating more superficial nodules with a finger through the port site incision and marking the palpated area before continuing with VATS resection [14]. A technique using radiological guided methylene blue for marking prior to breast microdochectomy has also been shown to have equal diagnostic accuracy compared to wire marking [15].

The need for accurate localization particularly for deeper lung nodules is essential for the success of VATS procedure for lung metastasectomy. The technique described is a cost effective, safe, and reliable method in marking lung nodules prior to resection. The method does not require expensive equipment described in other studies; nor does it run the risk of wire dislodgement. As with the studies from Lenglinger et al. in 1994 [5] and McConnell et al. in 2002 [6], no patients experienced any adverse effects of the methylene blue-autologous blood mixture and no major complications occurred.

Small pneumothorax is a common minor complication in any procedure where a needle passes through the pleura and would usually resolve spontaneously without further management; however, all these patients were undergoing immediate surgery. Piercing the pleura multiple times increases the risk of causing pneumothorax and therefore good planning prior to the procedure with a safe route for the needle to pass is essential [16]. One of the main risk factors for pneumothorax development is background chronic obstructive pulmonary disease and its associated complications, such as blebs and bullae [17]. Other risk factors for the development of pneumothorax include increasing patient age, increased depth of lesion, increased time of needle across the pleura, and traversing a fissure [16, 17]. Consideration also needs to be made of the motion of the lung with breathing and intermittent breath holds may be required during acquisition of images and advancement of the needle [16]. The incidence of pneumothorax is reported to range from 9% to 54% for lung nodule biopsy [16, 17], with an average of 20% [18], rising to 39% in patients with background emphysema [19]. Our pneumothorax rate of 7% with this technique is less than the aforementioned 9 studies and none lead to any complication during subsequent induction of general anaesthesia. None of the patients in this cohort had significant emphysema, bulla, or blebs.

An issue that we discovered is that even though only a small volume is injected, the dye can spread over a much larger area in the adjacent lung than simply the parenchyma surrounding the nodule to be marked; thus, as the injection is started, this needs to be at a slow and steady rate while withdrawing the needle back to the visceral pleural surface.

Clear communication with surgeons performing the subsequent metastasectomy is paramount and reconstructed multiplanar CT images of the nodule location aid their procedure planning. This and the discussion with the surgeon when there is the slightest concern when the marking is not exact will inevitably reduce failure rates and complications.
